# Two Decadal Experiences in Managing Combined Obstetric Vesicovaginal and Rectovaginal Fistulas: A Study From Northern Indian Tertiary Hospital

**DOI:** 10.7759/cureus.40198

**Published:** 2023-06-09

**Authors:** Vishwajeet Singh, Mohit Pandey, Jitender Yadav, Mohammad Rehan Akhtar, Mukul K Singh

**Affiliations:** 1 Urology, King George's Medical University, Lucknow, IND; 2 Radio Diagnosis, T. S. Misra Medical College & Hospital, Lucknow, IND

**Keywords:** obstetric, necrosis, martius flap, rectovaginal fistula, vesicovaginal fistula

## Abstract

Introduction: A retrospective study of 28 patients with obstetric combined vesicovaginal fistula (VVF) and rectovaginal fistula (RVF) treated at our centre throughout the last two decades (2002 to 2022) has been conducted.

Material and method: In 12 patients, a preoperative diverting colostomy was performed. Six patients had single-stage surgery (both VVF and RVF repair in the same operation) of which two cases required transabdominal repair and four required transvaginal repair.

Result: All single-stage repairs (n=6) were successful in curing urine and faecal incontinence. In 22 patients, VVF was corrected initially via the transvaginal method with Martius flap interposition, followed by RVF repair three months later. In 2/22 patients, there was a leak after RVF repair; therefore, proximal diverting colostomy was performed, and RVF repair was repeated after six months.

Conclusion: All cases had effective VVF and RVF repairs, and both urine and faecal incontinence were completely cured. This study suggests the collaborative engagement of a urologist and a surgical gastroenterologist results in an advantageous outcome for the surgical treatment of these intricate obstetric fistulas.

## Introduction

The obstetric fistula is due to the formation of an epithelised hole between the vagina and bladder (vesicovaginal fistula), the vagina and urethra (urethrovaginal fistula), the ureter and vagina (ureterovaginal fistula), and the vagina and rectum (rectovaginal fistula) [1]. These fistulae are usually isolated findings, but sometimes they can co-exist together, such as vesicovaginal and rectovaginal fistulas, which can co-exist in 1% to 23% of cases [2]. The presentation of vesicovaginal fistula (VVF) is urinary incontinence per vagina and that of rectovaginal fistula (RVF) is leakage of flatus and faeces through the vagina [3]. The association of VVF with RVF is characterised by a persistent offensive odour and leakage of urine with faeces leading to social stigma and ostracisation of affected women [4]. The basic mechanism for the formation of obstetric fistulae is ischemic necrosis. In prolonged obstructed labour, the foetal head puts prolonged compression over the vaginal and bladder wall soft tissues, leading to ischemia, necrosis, and the subsequent formation of VVF [5]. Similarly, prolonged compression of the foetal head over the soft tissues of the vaginal wall and rectum leads to the formation of RVF. The associated injury created during precipitous delivery and obstetric manoeuvres further leads to fistula formation [6]. The combined occurrence of these two devastating obstetric fistulae poses challenges to urologists and gastroenterologists. Additionally, nutritional status, anaemia, status of local tissue, history of previous repair, and associated co-morbidities are factors that affect the success of surgical reconstructions. The present study is a retrospective review of the records of patients who had combined obstetric VVF with RVF and underwent surgical repairs in our hospital, along with an analysis of their outcome results.

## Materials and methods

This retrospective study was carried out in the Department of Urology, King George’s Medical University, Lucknow, Uttar Pradesh, India. The institutional ethical committee approved the study with reference number 115th ECM 11A/P339. The study was in accordance with the Declaration of Helsinki. The retrospective review of records was done between 2003 and 2022. The patients (combined VVF and RVF) treated were included in this study. A total of 28 such patients had a history of surgical repair. The obstetric history and findings of clinical examinations were retrieved. The vaginal and rectal examinations, including the size and site of fistulae, were recorded. The history, including the place of delivery (hospital or domicile), associated perineal tear, and repair following childbirth were also recorded. In 18 patients, there was a history of third-degree (n=10) and fourth-degree (n=8) perineal tears, which were initially repaired and managed by obstetricians and surgeons before referral to our centre. A diverting colostomy was already performed by the treating surgeon in eight patients before referral to our centre, and additionally, in four patients, the colostomy was performed at our centre. The cystoscopic, vaginoscopic, and colonoscopic findings were noted. The findings of upper urinary tract imaging (ultrasound of kidney, ureter, and bladder), intravenous pyelogram (IVP), contrast-enhanced CT scan (CECT), abdomen, and pelvis were noted. The records also included the blood chemistries and tests for fitness for anaesthesia.

Diverting colostomy

The colostomy was done in cases of the infected vaginal mucosa, peri-anal infections, and failed previous repairs. Those with high rectal fistulas were also subjected to colostomies. Severe local infections and excoriations were present in eight patients. Two patients who had failed previous repairs done at another hospital were also subjected to colostomy. Overall, transverse colostomy was done in eight cases and sigmoid colostomy in four cases, respectively. Surgical gastroenterology consultation was taken before the colostomy (n=4), and all colostomies were performed by surgical gastroenterologists. About 10-12 weeks following colostomy, either VVF or RVF was repaired first (in two different operations), except in six cases in which both fistulae (VVF and RVF) were repaired in the same operation. In staged repairs, our preference was to first repair the vesicovaginal fistula. The rectovaginal fistula was repaired three months following successful VVF repair. In six cases, both VVF and RVF were repaired in the same operation.

Vesicovaginal fistula (VVF) repair

The transvaginal fistula repair was done in all patients in the modified Jackknife position. The cystoscopy and ureteric catheterization were done first in the lithotomy position in those cases where the ureteric orifice was close (within 1 cm) to the fistula. In all cases, a suprapubic cystostomy was done before putting the patient in the modified Jackknife position. A circumferential incision was made all around, about 0.5 cm from the fistula margin. The vaginal wall was dissected sharply and separated from the bladder wall, creating a plane between the bladder and vaginal walls. The bladder wall was closed by 3-0 polygalactin as an interrupted closure. A Martius fibrofatty vascularised flap was dissected from one side of the labia and placed through a submucosal tunnel created in the vaginal wall to the level repaired bladder as an interposition flap and tucked to the sutured line. The vaginal wall closed as an interrupted single layer of 3-0 polygalactin. A 16 F Foley catheter was placed pre-urethral and the balloon inflated to 10 ml. A small width corrugated drain was placed at the site of dissection of the Martius flap, and the labial skin was closed as a single layer by a silk or nylon interrupted suture. The vagina was packed with sterile cotton gauze soaked in 0.3% povidine iodine. The vaginal pack was removed after 24 hours, and the corrugated drain was removed 48 hours following the procedure. Bilateral ureteric catheters were removed after 24-48 hours, and continuous bladder drainage was ensured by suprapubic and urethral catheters. A cystogram was obtained three weeks following the procedure, and catheters were removed if there was no evidence of contrast extravasation. If there was some evidence of contrast extravasation, then catheters were kept for one to two weeks, and then a voiding trial was given.

Rectovaginal fistula (RVF) repair

Patients were placed in a lithotomy position. A circumferential incision was made all around, about 0.5 cm from the margin of the fistula. A sharp dissection was done to enter the plane between the vaginal and rectal walls. The rectal opening closed as an interrupted suture of 3-0 polygalactin. A second layer of peri-rectal fascia was closed with an interrupted suture. The torn levator muscle layer was brought in at the midline and loosely stitched together. A Martius labial fibrofatty vascularised flap was raised and brought through a tunnel created under the mucosa of the vagina to the rectal suture lines and tucked by polygalactin suture. The vaginal wall was closed by 3-0 polygalactin as an interrupted suture. A small width corrugated drain was placed in a place where the Martius flap was raised. The vaginal lumen is packed with loose sterile cotton gauze soaked in 0.3% povidine iodine and removed after 24 hours. The corrugated drain was removed after 24-48 hours. Patients are allowed oral liquid and a low residual diet once they pass flatus and faeces.

Repair of VVF and RVF in the same operation

This was done in six patients, and all cases had successful repair with a complete cure of urinary and faecal incontinence. Two patients had high RVF with VVF in whom transabdominal repair of VVF and RVF with omentum flap interposition was done in the same operation. In one patient, the left ureteric orifice was at the margin of the fistula, and ureteric reimplantation was done in the same operation. In four cases of low-lying fistulae (VVF with RVF), transvaginal repair of both fistulae was done in the lithotomy position. The VVF repair was done first, followed by the rectovaginal repair with Martius flap interposition in the same operation.

Colostomy closure

This was done after 12 weeks following the closure of the rectovaginal fistula. Following mechanical bowel preparation under general anaesthesia, an end-to-end bowel resection anastomosis was done. The patients were allowed oral liquids and a low residual diet once there was good bowel sound and patients passed flatus and faeces.

All patients are advised to maintain sexual abstinence for three months following VVF and RVF repairs. They also advised undergoing a lower-section caesarean section for future pregnancy and delivery.

## Results

The mean age of patients was 27.25 years, with a range of 19-35 years (Table 1). The youngest patient was just 19 years old, and she was primiparous with short stature (height 148 cm). Single-stage repair (closure of VVF and RVF in the same operation) was done in six patients (two patients by transabdominal route and four patients by transvaginal route), and two-stage repair was done in 22 cases, respectively. In 2/22 patients, the rectovaginal fistula closure leaked/failed following repair, but in both patients, the VVF was repaired first and was successful. In both patients, a diverting colostomy was performed following the failure of RVF repair. A repeat RVF repair was performed six months following the initial failed repair, and the outcome was successful. The colostomy was closed three months following RVF repair. All patients following surgical repair were continent for urine and faeces until the last follow-up. Twelve women became pregnant and delivered their next baby by lower segment caesarean section (LSCS) after a mean follow-up of 3.5 years (range: 2-8 years).

**Table 1:** Characteristics of Patients VVF: vesicovaginal fistula; RVF: rectovaginal fistula; VD: vaginal delivery, AVD: assisted vaginal delivery; LSCS: lower segment caesarean section; TVR: transvesical repair; TAR: transabdominal repair.

S.No	Age	Parity	Mode of childbirth	Characteristics of VVF	Characteristics of RVF	Previous VVF repair	Previous RVF repair	Colostomy	VVF repair at our centre	RVF repair at our centre	Single-stage/staged repair	Outcome of repair	Colostomy closure
1.	22	2	VD	Trigonal 2 cm	1.5 cm low rectal	No	No	Yes	TVR	TVR	Staged repair	Successful	After 3 months, successful
2.	25	1	VD	Trigonal 2 cm	1 cm low rectal	No	No	No	TVR	TVR	Staged repair	Successful	Colostomy not done
3.	28	2	VD	Trigonal 2 cm	1.5 cm low rectal	No	No	No	TVR	TVR	Staged repair	Successful	Colostomy not done
4.	32	3	LSCS	Supratrigonal 1.5 cm	1 cm high rectal	No	No	Yes	TAR	TAR	Single stage	Successful	After 3 months, successful
5.	30	2	VD	Bladder neck and proximal urethra 1.5 cm	1 cm low rectal	No	No	No	TVR	TVR	Staged repair	Successful	Colostomy not done
6.	29	1	VD	Trigonal 1.5 cm	0.8 cm low rectal	No	Failed repair	Yes	TVR	TVR	Staged repair	Successful	After 3 months, successful
7.	19	1	VD	Trigonal 2.5 cm	1 cm low rectal	No	No	Yes	TVR	TVR	Single stage	Successful	After 3 months, successful
8.	23	1	AVD	Trigonal 2 cm	0.5 cm low rectal	No	No	No	TVR	TVR	Staged repair	Successful	Colostomy not done
9.	25	1	VD	Trigonal 2.5 cm	2 cm low rectal	No	No	No	TVR	TVR	Staged repair	Failed	Colostomy not done
10.	30	2	VD	Trigonal 2 cm	1.5 cm low rectal	No	No	Yes	TVR	TVR	Staged repair	Successful	After 3 months, successful
11.	28	2	VD	Trigonal 2.5 cm	1 cm low rectal	No	No	Yes	TVR	TVR	Single stage	Successful	After 3months, successful
12.	25	1	AVD	Bladder neck and proximal urethra 1.5 cm	1 cm low anal	No	No	No	TVR	TVR	Staged repair	Successful	Colostomy not done
13.	31	3	VD	Trigonal 2 cm	0.8 cm low rectal	No	Failed repair	Yes	TVR	TVR	Staged repair	Successful	After 3 months, successful
14.	33	4	LSCS	Supratrigonal 2.5 cm with ureteric involvement	1.5 cm high rectal	No	No	Yes	TAR with ureteric reimplantation	TVR	Single stage	Successful	After 3 months, successful
15.	28	2	VD	Trigonal 1.5 cm	1 cm low rectal	No	No	No	TVR	TVR	Staged repair	Successful	Colostomy not done
16.	30	2	VD	Trigonal 2.5 cm	1 cm low rectal	No	No	No	TVR	TVR	Staged repair	Successful	Colostomy not done
17.	35	2	VD	Trigonal 2 cm	0.8 cm low rectal	No	No	No	TVR	TVR	Staged repair	Successful	Colostomy not done
18.	26	1	VD	Trigonal 2 cm	1 cm low rectal	No	No	Yes	TVR	TVR	Staged repair	Successful	After 3 months, successful
19.	28	1	VD	Trigonal 2 cm	0.8 cm low rectal	No	No	No	TVR	TVR	Staged repair	Successful	Colostomy not done
20.	22	1	VD	Trigonal 2 cm	2 cm low rectal	No	No	No	TVR	TVR	Staged repair	Failed	Colostomy not done
21.	24	1	AVD	Bladder neck and proximal urethra 2 cm	1 cm anal	No	No	Yes	TVR	TVR	Staged repair	Successful	After 3 months, successful
22.	25	1	VD	Trigonal 2 cm	0.8 cm low rectal	No	No	No	TVR	TVR	Staged repair	Successful	Colostomy not done
23.	28	2	VD	Trigonal 2 cm	1 cm low rectal	No	No	No	TVR	TVR	Staged repair	Successful	Colostomy not done
24.	27	1	VD	Trigonal 2 cm	1.5 cm low rectal	No	No	Yes	TVR	TVR	Single stage	Successful	After 3 months, successful
25.	30	2	VD	Trigonal 2 cm	1.5 cm low rectal	No	No	No	TVR	TVR	Staged repair	Successful	Colostomy not done
26.	25	1	VD	Trigonal 2 cm	0.8 cm low rectal	No	No	No	TVR	TVR	Staged repair	Successful	Colostomy not done
27.	29	1	VD	Trigonal 1 cm	0.8 cm low rectal	No	No	Yes	TVR	TVR	Single stage	Successful	After 3 months, successful
28.	26	1	VD	Trigonal 2 cm	2 cm low rectal	No	No	No	TVR	TVR	Staged repair	Successful	Colostomy not done

## Discussion

Obstetric trauma is the leading cause of vesicovaginal and rectovaginal fistulas formation in developing countries. The overall cases of obstetric vesicovaginal fistula (VVF) alone is more than 0.5 million across the world, rectovaginal fistula (RVF) alone is in 1% to 8% of cases, and combined vesicovaginal and rectovaginal fistulas co-exist in 1% to 23% of cases, respectively [2]. The mechanism of injury in obstetric fistula is pressure necrosis following prolonged obstructed labour, obstetric manoeuvres in precipitous delivery, and sometimes following elective abortions [3,4]. In the developed world, obstetric VVF and RVF are rare now, and in most cases, these fistulae are due to pelvic surgery, malignancy, radiotherapy, or a combination of the above factors [2-4,7].

Several important risk factors for developing obstetric fistulae have been reported in the literature, such as the presence of a skilled birth attendant, duration of labour and use of partograph, lack of prenatal care, height (less than 150 cm), elderly patients, lack of family planning, and other poorly defined additional factors [8]. These risk factors can simply be minimised by educating the community. The occurrence of obstetric fistulae and their consequences is devastating to patients and society. WHO recommendation is that by educating women and monitoring labour, the incidence of obstetric fistulae can be minimised. Ideally, the labour should be monitored on a partogram, but it is impossible to follow in domiciliary delivery [9]. In unsuccessful home delivery, patients reach the hospital in late and neglected stage. The cephalopelvic disproportion remains the major cause of obstructed labour in teenage and primiparous women. This can be prevented by antenatal and prenatal care [10]. Further delay in intervention in precipitous deliveries increases the time of compression of the mother’s soft tissue in pelvic organs such as the bladder and rectum between the foetal head and the mother's pelvic bones, leading to VVF and RVF with foetal death in the majority. All these together cause strong psychological setbacks for patients. All these factors, if taken care of, make combined obstetric VVF and RVF preventable [8,11].

In the present study, the majority of patients had low-lying fistulae. The transvaginal repair of vesicovaginal and rectovaginal fistulas, either in one sitting (single operation) or two different operative sessions (staged repair), can be done with Martius flap interposition. The association of VVF with RVF is categorised as complicated VVF, and the success rate for VVF repair in such cases has been reported at more than 86% [12,13]. Apart from the Martius vascular pedicle flap, the gracilis muscle can also be used as an interposition flap [14]. Transabdominal repair of VVF and RVF can be done in the same operation with a success rate of more than 87%. Transabdominal closure is recommended for supratrigonal fistulas with associated high rectovaginal fistulas. Additionally, ureteric reimplantation can be performed in the same operation. The omentum, peritoneal folds, or tinea epiploic are used as interposition flaps [15]. In the present study, in one case, transabdominal repair of VVF and RVF with omental flap interposition and left ureteric reimplantation was performed in the same operation.

RVF can be repaired by a range of surgical approaches, such as trans-anal, transvaginal, and transperineal repairs, with a success rate of 50%-90% [14-16]. Local advancement flaps such as the Martius flap and gracilis muscle interposition can be done to increase the success rate [17,18]. In the trans-anal approach, the fistula with scar tissue is excised first, followed by dissection of the rectovaginal septum to separate these two layers. The vaginal and rectal openings are then closed separately by absorbable sutures, and an interposition flap is inserted [19]. The transvaginal approach to repairing the rectovaginal fistula involves the excision of the fistula and scar tissue with multilayered tension-free closure of the rectal wall and perirectal fascia [20]. The torn levator muscle is approximated at the midline, and levatorplasty is performed. A Martius flap or gracilis muscle can be interpositioned, and then vaginal wall closure is performed by absorbable sutures. In an interesting and landmark study, Reisenauer reported on the transvaginal closure of the rectovaginal fistula with a success rate of 92.4% [16]. Transvaginal repair of VVF and RVF with an interposition flap has shown excellent results [12-14,19,21,22].

In transperineal closure, the rectum is separated from the vagina through a perineal incision. The rectal opening is closed, and the rectovaginal septum is strengthened by approximating the torn levator ani muscle. The vaginal opening is closed by an absorbable suture. An interposition flap can be interposed following rectovaginal septum reconstruction [23].

The diverting stoma or colostomy before VVF with RVF repair remains debated. The association of VVF with RVF usually involves severe obstetric injury with associated scarring and ischemia of surrounding tissue. Additionally, the failed previous repair, local infection, and excoriations are risk factors for the failure of the repair [24]. Following diverting colostomy, the fistula gets to rest, and there is a healing of unhealthy soft tissue with control of infection [25]. Patients without a diverting colostomy sometimes start taking less food in fear of the passage of faeces per vaginum, and if this habit persists longer, it leads to poor nutritional status (anaemia and hypoproteinemia). However, the colostomy causes great emotional and physical distress to the patients, so a routine colostomy should not be done; rather, it should be selectively indicated in cases of failed previous repair, high RVF, complex fistula, poor nutritional status, surgeon’s discretion with fear of poor wound healing, and unhealthy and infected local tissue around the fistula.

## Conclusions

Combined obstetric VVF and RVF are real challenges for urologists and gastroenterologists. Single-stage repair of VVF with RVF without a diverting colostomy can be done well. The diverting colostomy should be indicated only in selected cases. Staged repair definitely has a place in management and gives good results. In staged repair, the preference for repair either VVF or RVF first is based on the discretion of the urologist. The combined involvement of the urologist and surgical gastroenterologist gives a good outcome for the surgical repair of these complex obstetric fistulae.
